# Cooling Rate and Compositional Effects on Microstructural Evolution and Mechanical Properties of (CoCrCuTi)_100−x_Fe_x_ High-Entropy Alloys

**DOI:** 10.3390/e26100826

**Published:** 2024-09-29

**Authors:** Brittney Terry, Reza Abbaschian

**Affiliations:** 1 Department of Electrical and Computer Engineering (Materials Science and Engineering Program), University of California, Riverside, CA 92521, USA; 2 Department of Mechanical Engineering (Materials Science and Engineering Program), University of California, Riverside, CA 92521, USA; rabba@engr.ucr.edu

**Keywords:** HEA, cooling rate, LPS, hexagonal

## Abstract

This study investigates the impact of cooling rate and alloy composition on phase formations and properties of (CoCrCuTi)_100−x_Fe_x_ (x = 0, 5, 10, 12.5, 15) high-entropy alloys (HEAs). Samples were synthesized using arc-melting and electromagnetic levitation, followed by quenching through the use of a Cu chill or V-shaped Cu mold. Cooling rates were evaluated by measuring dendrite arm spacings (DASs), employing the relation DAS = k ɛ^−n^, where constants k = 16 and n = ½. Without Fe addition, a microstructure consisting of BCC1 + BCC2 phases formed, along with an interdendritic (ID) FCC Cu-rich phase. However, with the addition of 5–10% Fe, a Cu-lean C14 Laves phase emerged, accompanied by a Cu-rich ID FCC phase. For cooling rates below 75 K/s, alloys containing 10% Fe exhibited liquid phase separation (LPS), characterized by globular Cu-rich structures within the Cu-lean liquid. In contrast, for the same composition, higher cooling rates of 400–700 K/s promoted a dendritic/interdendritic microstructure. Alloys with 12.5–15 at. % Fe displayed LPS irrespective of the cooling rate, although an increase in uniformity was noted at rates exceeding 700 K/s. Vickers hardness and fracture toughness generally increased with Fe content, with hardness ranging from 444 to 891 HV. The highest fracture toughness (5.5 ± 0.4 K_IC_) and hardness (891 ± 66 HV) were achieved in samples containing 15 at. % Fe, cooled at rates of 25–75 K/s.

## 1. Introduction

The study of high-entropy alloys (HEAs) has spurred exploration into novel alloy combinations consisting of multiple principal elements in equiatomic or near-equimolar ratios [1,2,3,4,5,6,7,8]. In the current literature, these alloy combinations often give rise to simple crystal structures (BCC, FCC, and HCP) rather than competing intermetallic compounds [1,2,7,9,10,11,12,13,14]. However, alongside the development of solid solution phases, research has highlighted instances of potential liquid phase separation (LPS) and other minor phases within the microstructure of certain HEA systems [2,3,5]. These phases significantly influence mechanical properties, notably hardness and fracture toughness, owing to their role in shaping the microstructure. Adjustments in cooling rate [2,7] and/or incorporation of minor additive elements [3,5] have been demonstrated to refine the micro-structure of HEAs, affecting both phase composition and morphology of constituent phases [7,15].

The known properties of as-cast single-phase HEA states have been emphasized by several researchers, as summarized by Tsai and Yeh [6]. However, insufficient emphasis has been placed on the development and characterization of complex multi-phase HEAs. For example, a dendritic/interdendritic dual-phase microstructure has been developed at cooling rates of approximately 10–20 K/s in the AlCoCrCuFeNi HEA [16]. In contrast, a polycrystalline microstructure has been observed at cooling rates of 106–107 K/s, along with a decrease in elastic modulus, as discovered by Singh et al. [16]. This difference in phase development is primarily attributed to the change in cooling rate, a phenomenon also observed in the Al_0.5_CoCrFeNi HEA. Lin et al. investigated the as-cast Al_0.5_CoCrFeNi HEA, revealing an FCC solid solution phase and a Vickers hardness of 247 HV [17]. Conversely, Xiong et al. studied cooling effects on the same alloy, which resulted in a BCC + FCC + B2 microstructure, with an increased Vickers hardness of 393 HV at cooling rates of 0.02–0.03 K/s. When the cooling rate increased to over 75 K/s, a subsequent rise in hardness to 547 HV was observed [7,18]. Expanding the analysis of Al_0.5_CoCrFeNi to include additional cooling parameters caused the development of an additional phase and improved hardness [7,17,18].

The prevailing literature currently emphasizes HEAs that form BCC or FCC crystal structures [19], with limited attention given to the alloys forming hexagonal phases [4,12,20]. According to Zhu et al., FCC phases are mentioned in 56% of the current literature, and BCC phases are discussed in 43% of literature, with HCP phases accounting for the remaining 1% reported [20]. Arguments as to why hexagonal HEAs are less studied include the development of low fracture toughness and its tendency to form either ordered hexagonal C14 Laves or disordered HCP phases [2]. However, hexagonal phases are also known to exhibit remarkable high-temperature strength due to their elevated melting points, hardness, yield strength, strength-to-weight ratios, creep resistance, thermal stability, and superior wear resistance [1,2,3,21,22,23]. The densities of these alloys were measured at around 7.05 g/cm^3^ for similar HEAs containing Mn instead of Fe [12].

The presence of the hexagonal C14 Laves phase with its exceptional hardness has been observed in various HEA systems, such as the (CoCrCuTi)_100−x_Mn_x_ (x = 0, 5, 10, 15, 20) HEA [3]. This investigation studied the influence of Mn on the formation of a majority hexagonal microstructure, along with changes to mechanical properties [3]. Upon observation, alloys containing 10 at. % Mn or less exhibited a BCC dendritic phase and FCC interdendritic matrix. Above 10 at. % Mn, the development of a C14 Laves phase was observed, alongside an interdendritic Cu-rich FCC matrix. The Vickers hardness of the C14 Laves phase in Co_22_Cr_18_Cu_20_Mn_16_Ti_24_ was observed at 996.6 ± 57.2 HV, though the soft FCC phase negatively impacted the overall hardness, decreasing to 480.2 ± 21.4 HV.

A_lx_CoCrCuFeNi alloys with x < 0.6 have been shown by Munitz et al. [1] to have FCC crystal structures, with ductility ranging from 20% to 60%. These alloys also tend to exhibit significant work hardening but have relatively low hardness values, ranging from 100 to 200 HV [1]. In comparison, Demirow et al. [5] reported HCP + FCC dendrites in Co_23_Cr_22_Cu_3_Ti_28_Fe_24_ alloys, where the overall hardness was 784 ± 24 HV. Another investigation showed the influence of varying additions of Zr on phase formations and mechanical properties of (CoCrCuTi)_100−x_Zr_x_ (x = 0, 3, 5, 7, 10) [21]. They showed that without the addition of Zr, the alloy consisted of a main FCC phase and Cu-rich interdendritic FCC phase. However, adding 3 at. % Zr caused the FCC interdendritic phase to transform into an interdendritic C14 Laves phase, enhancing hardness. At 10 at. % Zr, the microstructure transitioned from a uniform dendritic morphology to randomly ordered dendrites within a dual-phase C14 + FCC microstructure. The addition of Zr also resulted in increased compressive strength due to C14 Laves phase formation, with 10 at. % Zr yielding the highest value at 668 ± 10.6 MPa. 

The phase formations of the (CoCrFeNi)(NiAl)(Nb_x_) HEA further revealed the development of a C14 Laves phase when Nb was added [22]. (CoCrFeNi)_87_(NiAl)_10_Nb_3_ displayed a divorced eutectic microstructure, consisting of a dual C14 + FCC phase, attributed to low Nb content. As Nb content increased to 8 at. %, a shift was observed towards a triple-phase eutectic micro-structure, comprising FCC + C14 + B2 phases. When increased further to 10.5 at. % Nb, samples fully developed a triple-phase eutectic microstructure. Samples containing 3 at. % Nb developed a fracture strain of above 35%, yet the increase in Nb content to 10.5 at. % lowered the fracture strain to 10.8%.

While most HEAs have predominantly featured transition metals, it is noteworthy that HCP phases have been observed in lanthanide-based HEAs. In the case of the TaNbHfZrTi HEA, a Hf/Zr-rich hexagonal phase formed when annealed at temperatures ranging from 500 to 900 °C [24]. This phase transformation resulted in an increased hardness from approximately 384 to 528 HV after annealing at 500 °C for 24 h. Additionally, the arc-melted GdHoLaTbY HEA displayed a single-phase HCP structure characterized by irregular grain structures [25]. Mechanical properties indicated a compressive yield strength of 108 MPa, a maximum compressive fracture strength reaching 880 MPa, and a plasticity level of 21.8%. Notably, the hardness was measured at 96 HV, a value considerably lower than that typically observed in CoCrCuTi-based alloys.

Most hexagonal phases are known for their superior hardness [26], though their comparatively lower fracture toughness limits their applicability when compared to FCC and BCC materials [2,3]. Nonetheless, dual-phase FCC and BCC + HCP microstructures may address these challenges, as stated by Chen. et al. [27]. A study by Farjam et al. [28] furthered this claim by studying Cantor alloy (CoCrNiFeMn) to improve the strength–ductility trade-off. When the concentrations of Co, Fe, and Mn were changed, a dual-phase FCC + HCP microstructure developed in the Co_50_Cr_20_Ni_20_Fe_5_Mn_5_ HEA. During tensile deformation, the alloy exhibited a synergistic effect of plasticity induced by phase transformation (TRIP) and plasticity induced by twinning (TWIP), which enhanced its ductility. This led to improved properties, as demonstrated by a yield strength of 502 MPa, which exceeds the yield strength of 405 MPa reported for the referenced CoCrNiFeMn HEA [28]. Furthermore, the ultimate tensile strength increased to 1002 MPa, surpassing the referenced HEA’s ultimate tensile strength of 766 MPa [28].

In the as-cast state, the (CoCrCuTi)_100−x_Fe_x_ (x = 10, 15, 20) HEAs were shown to exhibit a C14 Laves phase and secondary Cu-rich FCC phase [5]. However, the presence of elongated C14 Laves dendrite arms limited the potential benefits from the interdendritic matrix, impeding the ability of the FCC matrix to arrest crack propagation. Furthermore, variations in the cooling rate have been shown to exert influence on phase formation and mechanical characteristics of HEAs [2,9,16,29,30,31,32,33,34,35,36], with Vickers hardness shown to increase with the cooling rate [37]. Additional classical thermodynamic parameters, such as mixing enthalpies, were calculated for various alloys by Demirow et al. [12], with a specific focus on CoCrCuTiFe systems and related compositions. For CoCrCuFeTi alloys, the mixing enthalpy (∆Hmix) was found to be −5.8 kJ/mol, with a valence electron concentration (VEC) of 7.6. This study aims to investigate changes in microstructural and mechanical characteristics of (CoCrCuTi)_100−x_Fe_x_ (x = 10, 12.5, 15) HEAs, documenting the phase evolution in these multicomponent systems as the cooling rate and/or composition changes.

## 2. Materials and Methods

Alloys consisting of (CoCrCuTi)_100−x_Fe_x_ (x = 10, 12.5, 15 at. %) were prepared from raw elements with purities of Co, Cr, Cu, Ti, and Fe at ≥99.9%, supplied by Alfa Aesar (Ward Hill, MA, USA). The arc melter was pumped to a vacuum level below 10⁻^3^ Pa, and the weighed components were arc-melted five times (flipped four times) in a Ti-gettered atmosphere. Subsequently, some arc-melted samples were vertically cross sectioned normal to the Cu hearth for microstructural analysis, while others were remelted and solidified using electromagnetic levitation.

**Figure 1:**
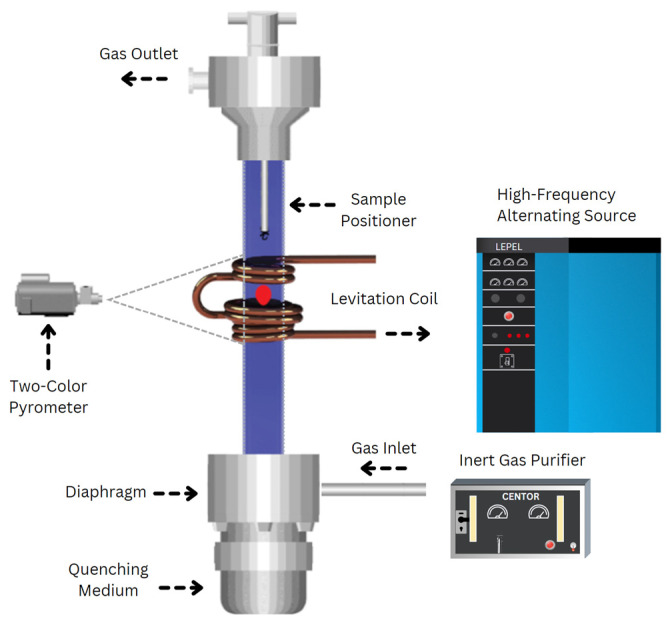
Schematic of electromagnetic levitation processing, illustrating a magnetically levitated sample enclosed within a quartz tube.

The experimental setup for electromagnetic levitation (EML) processing is illustrated in Figure 1. The setup included a levitation coil connected to a 20-kW generator operating within a frequency range of 300 kHz to 8 MHz, a temperature measurement and recording system, and various quenching media. A glass tube positioned within the coil facilitated the passage of an inert gas stream containing high-purity argon (99.999%) and helium (99.999%) to shield the sample from oxidation and regulate its temperature. A Metis M3 2-color pyrometer was utilized for temperature measurements of the levitated samples. The pyrometer reading has been indicated by the manufacturer to be accurate to +−0.5%. Reading rates changed from 100–300 readings per second. The melting point of the samples, as determined by the pyrometer, served as a reference for assessing its supercooling or superheating. During levitation melting, samples were introduced into the coil using a copper sample holder, which was removed once the solid sample began levitating. The percentage of power and gas flow were then adjusted to melt and superheat the sample. At a desired temperature, samples were dropped and quenched in a V-shaped copper mold or copper chill positioned below the levitation chamber.

To analyze the microstructures, samples were first mounted and polished using standard techniques. Subsequently, the polished samples were etched in a 5% nitric acid solution (nital) for 5 to 15 s, supplied by Alfa Aesar (Ward Hill, MA, MA). High-resolution images were captured using Canon EOS Utility 3.18.11 and a Nikon Eclipse LV 100D-U Microscope (Melville, NY, USA). Backscattered electron images (BSEs) were captured using a TESCAN VEGA3 SBH Scanning Electron Microscope (SEM) (Tempe, AZ, USA), equipped with energy dispersive spectroscopy (EDS) capabilities. The crystal structures of the developed phases were analyzed using a PANalytical Empyrean Series 2 X-ray Diffraction (XRD) instrument (Worcestershire, UK). This instrument functioned within a 2θ range from 20° to 100°, with a step size of 0.02° and duration of 40 s per step. Vickers microhardness tests were conducted using a Phase II Micro Vickers Hardness Tester (Upper Saddle River, NJ, USA), applying a load of 1 kg to the microstructure for 15 s. The average microhardness was calculated by determining the mean value from measurements obtained from 10 indentations.

## 3. Results and Discussion

### 3.1. Cooling Rate (K/s) Calculation 

**Figure 2:**
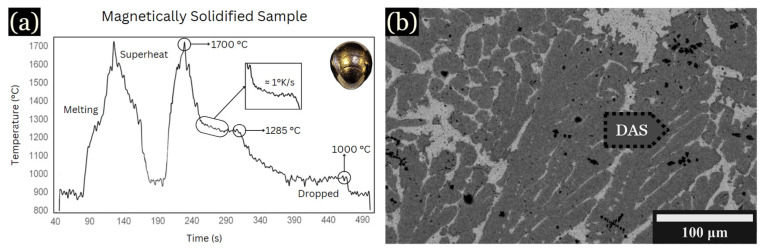
BSE images and graph of samples magnetically solidified. (**a**) Time vs. temperature graph. (**b**) Large, hexagonal dendrites shown for 10 at. % Fe, developed in the outer region of the sample at 1 K/s.

To determine the cooling rate versus DAS measurements, controlled cooling during levitation was utilized. An example is shown in Figure 2a, cooled at 1 K/s. The peak labeled “superheat” occurred at approximately 1700 °C, corresponding to the fully molten state of the sample. Due to fluctuations in the levitation sample temperature, the exact effect of iron additions on the liquidus temperatures were not determined, but they were near the liquidus temperature shown in Figure 2. Upon cooling, another notable peak at around 1285 °C indicates the initiation of solidification of the primary dendrites, while the peak around 1000 °C is associated with the solidification of an interdendritic Cu-rich phase, shown in Figure 2b. 

The average spacing for this sample was found to be ~17 µm. Using DASs along with the known cooling rate of 1 K/s, the constant k value was determined using Equation (1) [2]:

DAS = k ε^−n^      = 16 ε^−1/2^ (1)

Here, DAS is measured in micrometers (μm), with k being a constant for a particular alloy, ε representing the cooling rate in Kelvins per second (K/s), and n being typically within the range of ½ for primary DAS [2,38,39]. The value of k was determined to be 16, which agrees with previous measurements [5]. The above equation was used to determine cooling rates for arc-melted and quenched samples.

### 3.2. Microstructural Analysis of Arc-Melted (AM) Samples

**Figure 3:**
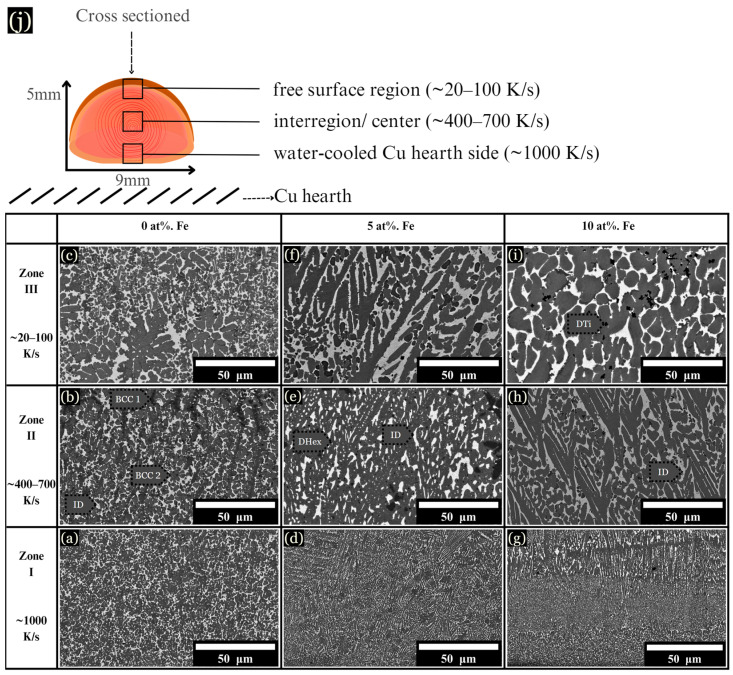
BSE images of microstructural changes as the cooling rate increases from ~20–1000 K/s. (**a**–**c**) 0 at. % Fe, (**d**–**f**) 5 at. % Fe, and (**g**–**i**) 10 at. % Fe, each corresponding to the specified cooling rates. (**j**) Schematic of a cross sectioned arc-melted sample, along with corresponding cooling rates.

The microstructure of arc-melted samples revealed different morphologies depending on the composition and distance from the copper substrate. These changes are described in three arbitrarily chosen zones for each sample, as schematically shown in Figure 3j, along with their corresponding cooling rates: (I) The initial solidification region, designated as the chill zone (up to ~75 μm from the hearth), formed where rapid solidification occurred due to contact with the Cu hearth. This zone developed fine and uniform dendrites, with a measured cooling rate of ~1000 K/s (Figure 3a,d,g). (II) Situated centrally within the sample, zone II solidified about 2 mm from the chill zone, with cooling rates ranging from ~400–700 K/s (Figure 3b,e,h). (III) Located up to ~1 mm from the top of the sample, zone III solidified last and experienced the slowest rate of solidification, with a cooling rate calculated between ~20–100 K/s (Figure 3c,f,i).

The base CoCrCuTi alloy, depicted in Figure 3a–c, revealed two dendritic phases labeled BCC1 and BCC2, alongside a Cu-rich interdendritic ID phase. In a comparative study, Demirow et al. [5] studied the CoCrCuTi alloy and provided the EDS data referenced in Table 1, along with a Vickers hardness of 444 ± 15 HV. BCC1 dendrites had a composition of Cr at 85.9 at. %, while BCC2 dendrites were composed of Co at 37.0 at. % and Ti at 37.9 at. %. BCC2 dendrites exhibited larger, blocky morphologies, as depicted in Figure 3c. Finally, the FCC interdendritic phase, labeled ID, solidified between the BCC dendrites, with a composition of Co (6.3 at. %), Cr (1.9 at. %), Cu (86.0 at. %), and Ti (5.9 at. %).

**Figure 4:**
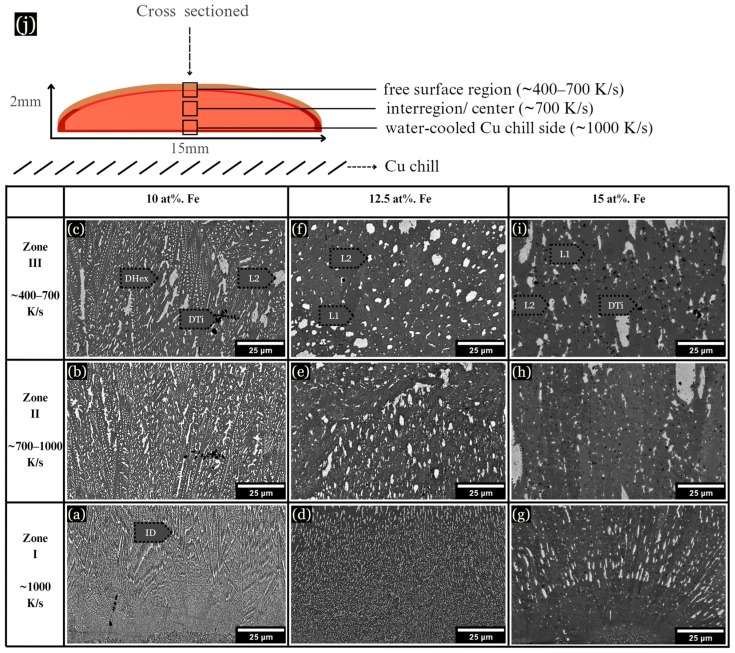
Samples were cast onto a Cu plate while molten, forming three main regions of solidification. (**a**–**c**) 10 at. % Fe microstructure from the chill zone and the center. (**d**–**f**) 12.5 at. % Fe sample, showing evidence of LPS. (**g**–**i**) 15 at. % Fe sample, with large L2 globules. (**j**) Schematic of samples quenched on a Cu chill.

**Figure 5:**
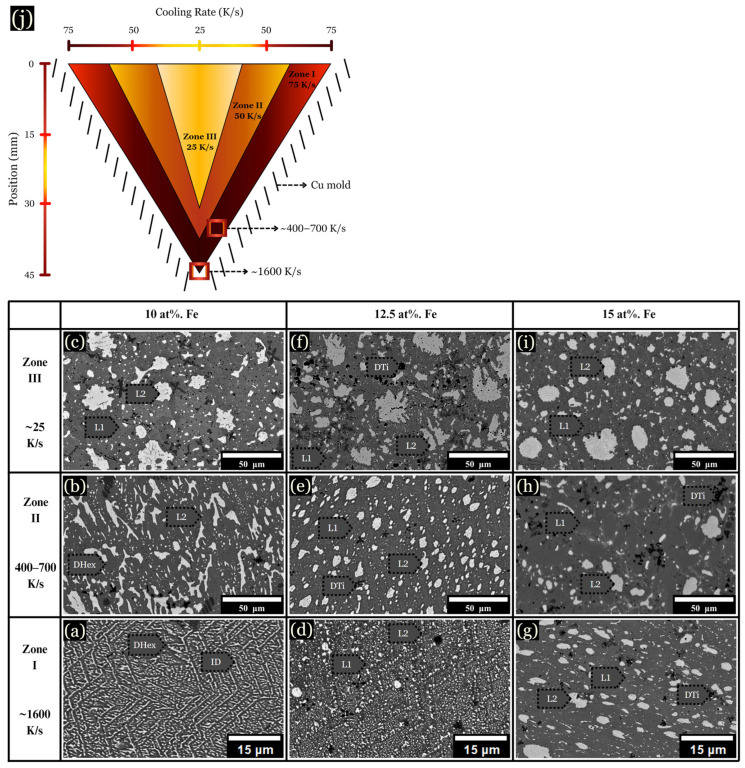
BSEs of arc-melted sample microstructures. (**a**–**c**) Alloys containing 10 at. % Fe are shown. (**d**–**f**) Images of 12.5 at. % Fe with hexagonal dendrites. (**g**–**i**) Microstructure of 15 at. % Fe. (**j**) Schematic of V-shaped Cu mold with corresponding cooling rates.

Upon the addition of 5 at. % Fe, the solidification process mirrored that of the base alloy, with fine dendrites observed near the chill zone and larger dendrites present in zone III (Figure 3f). However, instead of BCC phases, a hexagonal C14 Laves phase formed (DHex), surrounded by an interdendritic Cu-rich phase. Near the chill zone, with a DAS of ~0.5 μm and a cooling rate of ~1000 K/s, elongated hexagonal dendrites were observed growing upward from the chill. These hexagonal dendrites, characterized by arms at a 60° angle from the primary dendrite, were enveloped by the interdendritic matrix. Dark Ti-rich or Cr-rich secondary phases were dispersed across the microstructure, as shown in Figure 3, Figure 4 and Figure 5. At 10 at. % Fe, elongated DHex dendrites transitioned into the coarser dendrites observed from zone I to zone III. As evidenced by the augmented Cu-rich phase and broken DHex dendrite arms, Cu is rejected as the cooling rate decreases, shown in zone III for 10 at. % Fe (Figure 3i). Beyond 10 at. % Fe, evidence of LPS was observed in all zones, forming large Cu-rich globular structures, as described later. 

### 3.3. Microstructural Analysis of Quenched Levitated Samples

Samples levitated and dropped onto Cu substrates while molten solidified into a pancake shape of ~2 mm × 15 mm. These samples can be described in three arbitrary zones similar to arc-melted samples, as shown schematically in Figure 4j. In the chill zone at ~1000 K/s, the samples displayed small dendrites emanating from the chill plate (Figure 4a,d,g). As observed, 10 at. % Fe developed a Cu-lean DHex phase and Cu-rich ID phase from ~400–1000 K/s (Figure 4b). At 10 at. % Fe, dendrite coarsening became evident as the cooling rate decreased (Figure 4c). However, at 12.5 at. % Fe, the microstructure changed drastically, displaying LPS, as shown in Figure 4d–f. LPS is characterized by the presence of globular Cu-rich L2 structures (white-colored) within a gray-colored L1 liquid. With the addition of more Fe, the L2 globules became larger, as shown for 15 at. % Fe samples (Figure 4g–i). Note that LPS is very fine-scaled at faster cooling rates near the chill zones in Figure 4d,g.

When the cooling rate is approximately 1 K/s, the microstructure shows two distinct morphologies corresponding to liquid phase separation L1 and L2. For the alloy with 10 at. % Fe, L1 exhibits a relatively homogeneous distribution of Co (32.7 at. %), Cr (23.9 at. %), Ti (27.5 at. %), and Fe (12.7 at. %), with Cu being notably underrepresented (3.2 at. %). In contrast, L2 is characterized by a predominant concentration of Cu (94.2 at. %), which preferentially segregated into Cu-rich globules. This pattern remains consistent across alloys with 12.5 and 15 at. % Fe, although L1 reflects minor changes in Cr and Ti levels. As the cooling rate was increased to 50–150 K/s, L2 continued to be Cu-rich (94.5 at. %), indicating a strong tendency for Cu segregation, similar to what is observed in eutectic systems. Similar trends are observed in alloys with 12.5 and 15 at. % Fe. 

**Figure 6:**
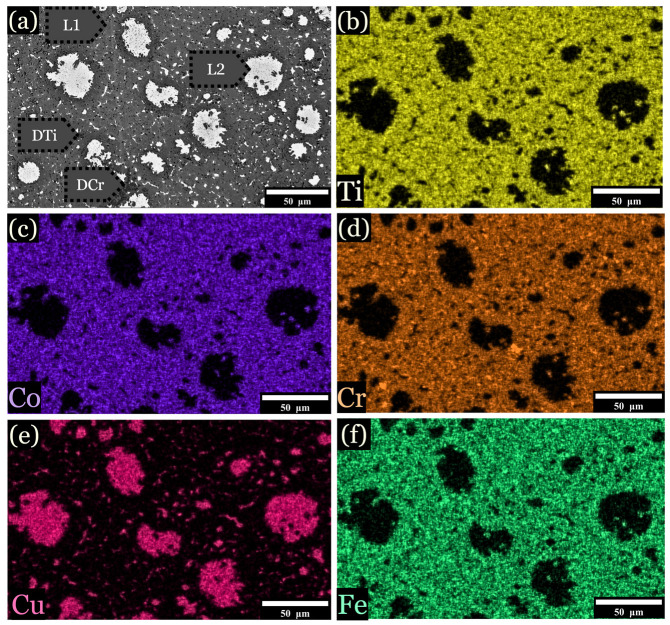
EDS imaging of the 12.5 at. % Fe sample. Samples were subjected to a cooling rate of approximately 25 K/s, is shown for specimens quenched into a V-shaped Cu mold. (**a**) Overall image, (**b**) distribution of titanium across the microstructure, (**c**) distribution of cobalt, (**d**) distribution of chromium, (**e**) distribution of copper, and (**f**) distribution of iron.

Figure 5 overviews the development of the microstructure as Fe increases, along with cooling rate influence. 10 at. % Fe developed small, uniform hexagonal dendrites surrounded by a Cu-rich ID phase, with DAS of ~0.4 μm. Upon reduction of the cooling rate to 25–75 K/s, the hexagonal dendrites were replaced by LPS, as shown in Figure 5f and Figure 6a–f, consisting of a Cu-lean L1 phase and Cu-rich L2 phase. The diameter of L2 globules ranged from 0.5–35 μm (Figure 5c,f,i). Similar LPS was observed in 12.5 and 15 at. % Fe (Figure 5d–f), though microstructures formed small, uniformly dispersed L2 globules.

**Figure 7:**
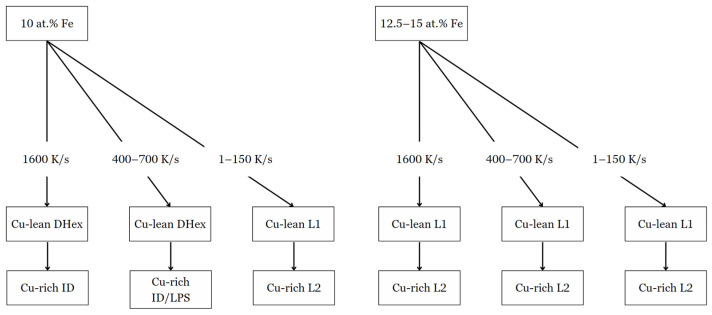
Phase formation diagram of 10–15 at. % Fe samples when subjected to specific cooling rates.

When the cooling rate increased to 400–700 K/s, signs of LPS began to disappear in 10 at. % Fe (Figure 5b,e,h), with L2 developing interdendritically. The microstructure transitions to a Cu-lean DHex phase and an ID or LPS Cu-rich region. In the alloy with 10 at. % Fe, the DHex phase demonstrated a more balanced distribution of the primary elements, with compositions of Co (30.7 at. %), Cr (26.0 at. %), Ti (26.6 at. %), and Fe (13.2 at. %), with low Cu content (Table 2). The Cu-rich region showed high Cu concentrations (95.3 at. %). 12.5 and 15 at. % Fe alloys exhibited similar phase behavior, with slight increases in Fe content in the DHex phase, but the basic segregation trend of Cu in the ID/LPS region remained unchanged. The phase formation and transformation sequence depended on both composition and cooling rate, as indicated in Table 1, Table 2, Table 3 and Table 4, and shown in Figure 7. 10 at. % Fe alloys from Dhex + ID phases at high cooling rates. However, at lower cooling rates, the phase sequence shifts to L1 + L2. In comparison, for 12.5 and 15 at. % Fe samples, LPS formed at all cooling rates. Table 3 and Table 4 give compositions of alloys containing 12.5 and 15% Fe, respectively, as described below.

At high cooling rates (400–700 K/s), the microstructure transitioned to a DHex phase and an interdendritic or low-phase segregation (ID/LPS) region. The DHex phase demonstrated a more balanced distribution of the primary elements, with compositions of Co (30.7 at. %), Cr (26.0 at. %), Ti (26.6 at. %), and Fe (13.2 at. %). The Cu content remained relatively low (3.4 at. %). The ID/LPS region, however, showed a markedly high Cu concentration (95.3 at. %), indicating that even at higher cooling rates, Cu segregation persists, with low solubility of Cu observed. At the highest cooling rate studied (~1600 K/s), the DHex phase composition shifts, with a notable increase in Cu for 10 and 12.5 at. % Fe. The ID phase, however, decreased in Cu content (64.1 at. %), alongside an increase in Co (11.9 at. %), Cr (8.1 at. %), Ti (11.6 at. %). 

The incorporation of Fe into the CoCrCuTi system significantly enhanced the phase complexity, with Fe concentrations in the L1 phase varying from approximately 10 to 19 at. % depending on both the alloy’s Fe content and the imposed cooling rates. At a cooling rate of 1600 K/s, L1 formed with an increased Cu content as the Fe concentration rose from 10 to 12.5 at. % (Table 3), causing the Cu content within L1 to increase from ~3% to over 21%. The reduced Cu rejection in L1 indicates that this phase was able to stabilize itself at the highest cooling rate, 1600 K/s, which contributed to the increase in fracture toughness, as shown in Table 5. At lower cooling rates, the Cu content remained approximately the same for Fe concentrations of 10 to 15 at. %, but at 1600 K/s, L1 experienced a notable increase in Cu content, as indicated in Table 2, Table 3 and Table 4. Concurrently, the Cu content in L2 decreased, with 10 at. % Fe developing the lowest Cu concentration (~67 at. %), compared to 92.7 at. % Cu in the 15 at. % Fe alloy.

### 3.4. Vickers Hardness 

**Figure 8:**
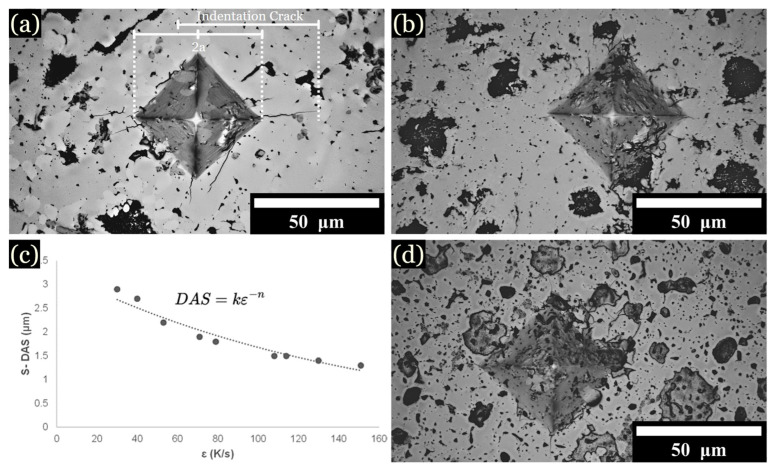
Optical imaging of Vickers hardness indentations: (**a**) Cu mold cast sample at 10 at. % Fe. (**b**) Cu mold cast sample at 15 at. % Fe. (**c**) DAS equation and graph. (**d**) Cu mold cast sample at 12.5 at. % Fe.

Vickers hardness (HV) values were measured for samples cooled from all zones. The specific influence of individual phases or their compositions on the mechanical properties will be investigated later. At 1 kg of force, the diamond tip caused cracks to propagate from the corners of the diamond indent (Figure 8a–d). Increasing the Fe content generally resulted in higher overall hardness values. The hardness of L1 was measured to be between 900 to 1100 HV for 10 to 15 at. % Fe samples, yet the hardness of L2 was noted to be below 300 HV for most samples. Slower cooling rates favored larger and more heterogeneous microstructures, resulting in decreased average hardness values. For instance, at 1 K/s, the hardness values decreased to 598 ± 129 HV for 10 at. % Fe, 683 ± 173 HV for 12.5 at. % Fe, and 623 ± 191 HV for 15 at. % Fe. Higher cooling rates favored uniformed dendrites that enhanced hardness. For instance, at 1600 K/s, the average hardness values were 802 ± 22 HV for 10 at. % Fe, 877 ± 35 HV for 12.5 at. % Fe, and 827 ± 53 HV for 15 at. % Fe (Table 5). As the Fe content increased, the greater scatter in hardness values reflected the increased heterogeneity across the microstructure. Rapid cooling rates favored uniformity across microstructures, contributing to higher average hardness values, as noted in Table 5. The structural transformation corresponding to a noticeable increase in hardness by varying cooling rates was also documented by Lin et al. [17] and Xiong et al. [7]. Additionally, the segregation patterns and microstructural refinement closely mirror the behaviors described in literature [2,7,29,34]. 

### 3.5. Fracture Toughness

At 1 kg of force, the diamond tip caused cracks to propagate from the corners of the diamond indent (Figure 8a). The crack length was used to calculated fracture toughness according to the following equation (Equation (2)) [23,40,41]:

KIC = 0.16 (c)^−3/2^ H√a, (2)

KIC = Fracture toughness (MPa√m),
c = Average crack length,
a = Half of the average diagonal length of the Vickers marks (microns),
H = Vickers hardness (MPa).

Generally, it was observed the addition of Fe increased fracture toughness, while cooling rate decreased this value. For instance, at 1600 K/s, 10 at. % Fe developed fracture toughness of 2.8 ± 0.1 KIC, while 15 at. % Fe was measured at 3.9 ± 0.3 KIC (Table 5). The addition of Fe was observed to increase fracture toughness due to formation of L1 + L2. At the same time, the high cooling rate could cause more residual stresses within the sample, which can reduce fracture toughness. The influence of residual stresses on fracture toughness has been given by Samuel et al. [42] for crack formation and propagation in steels. Samples cooled at 1000 K/s display this increase, where 10, 12.5, and 15 at. % Fe developed a fracture toughness of 1.7 ± 0.1 KIC, 1.7 ± 0.4 KIC, and 1.9 ± 0.6 KIC, respectively. At cooling rates of 400–700 K/s, fracture toughness values 10, 12.5, and 15 at. % Fe were 2.7 ± 0.6 K KIC, 3.3 ± 0.4 KIC, and 2.9 ± 0.5 KIC, respectively. At 25–75 K/s, the 15 at. % Fe developed the highest fracture toughness among compositions, found to be 5.5 ± 0.4 KIC. When cooled at 1 K/s, the fracture toughness values were similar for 10, 12.5, and 15 at. % Fe 3.9 ± 0.3, 4.1 ± 0.2, and 4.1 ± 0.3, respectively.

## 4. Summary

1. The influence of composition and cooling rate on the microstructural development and properties of (CoCrCuTi)_100−x_Fe_x_ (x = 0, 5, 10, 12.5, 15) high-entropy alloys (HEAs) were investigated utilizing arc-melting and EM levitation-melting, followed by quenching against a copper substrate or chill mold.
2. Arc-melted samples with no iron addition revealed BCC1 + BCC2 phases, followed by an interdendritic FCC Cu-rich phase. However, with the addition of 5 to 15% Fe, a Cu-lean C14 Laves phase emerged, accompanied by an interdendritic Cu-rich FCC phase.
3. At 10 at. % Fe, lower cooling rates (1–75 K/s) resulted in LPS, leading to the formation of Cu-lean L1 and Cu-rich L2 liquid phases. Increasing the cooling rate to ~400–700 K/s, the microstructure transformed into Cu-lean dendrites, with an interdendritic Cu-rich phase. However, coarse dendrites developed with a DAS of ~0.6–0.8 μm, surrounded by the Cu-rich liquid that was rejected and solidified at the end. At ~1000–1600 K/s, fine dendrites formed with a DAS of ~0.4–0.5 μm, alongside an interdendritic Cu-rich phase.
4. Alloys with 12.5 to 15 at. % Fe developed LPS regardless of cooling rate. However, higher cooling rates resulted in a more uniform distribution of L2 throughout the microstructure.
5. With the incorporation of Fe up to 15 at. % in the CoCrCuTi alloy, Vickers hardness increased from 444 to 891 HV when the hexagonal Laves C-14 phase was present.
6. Rapid cooling rates generally enhanced hardness due to the formation of more-refined microstructures. However, fracture toughness decreased as the cooling rate increased. The highest Vickers hardness (891 ± 66 HV) and fracture toughness (5.5 ± 0.4 K_IC_) values were observed in 15 at. % Fe samples at 25–75 K/s.

## Figures and Tables

**Table 1:** EDS analysis of CoCrCuTi samples, measured by Demirow et al. [5].

ɛ (K/s)	Phase	Co	Cr	Cu	Ti	Fe
	Nominal	25	25	25	25	-
**~1000 K/s**	BCC_1_	6.9	85.9	1.2	6.0	-
	BCC_2_	37.0	14.5	10.6	37.9	-
	ID	6.3	1.9	86.0	5.9	-

**Table 2:** EDS analysis of microstructural features (CoCrCuTi)_90_Fe_10_ samples.

ɛ (K/s)	Phase	Co	Cr	Cu	Ti	Fe
	Nominal	22.5	22.5	22.5	22.5	10
**~1 K/s**	L1	32.7	23.9	3.2	27.5	12.7
	L2	2.6	0.8	94.2	1.6	0.8
**50–150 K/s**	L1	30.8	33.2	3.4	18.9	13.7
	L2	2.1	1.2	94.5	1.4	0.8
**400–700 K/s**	DHex	30.7	26.0	3.4	26.6	13.2
	ID/LPS	2.2	0.8	95.3	1.5	0.3
**~1600 K/s**	DHex	26.8	18.1	20.9	24.0	10.4
	ID	11.9	8.1	64.1	11.6	4.3

**Table 3:** EDS data of microstructural features in (CoCrCuTi)_87.5_Fe_12.5_ samples.

ɛ (K/s)	Phase	Co	Cr	Cu	Ti	Fe
	Nominal	21.88	21.88	21.88	21.88	12.5
**~1 K/s**	L1	30.2	24.7	2.5	26.2	16.5
	L2	0.8	1.0	97.2	0.7	0.4
**~25 K/s**	L1 + L2	23.69	20.32	22.59	20.91	12.49
**25–75 K/s**	L1	27.9	26.1	4.5	25.4	16.1
	L2	0.8	0.8	95.8	2.2	0.4
**400–700 K/s**	DHex	27.8	25.6	6.7	23.4	16.5
	ID	2.0	1.5	93.7	2.0	0.7
**~1600 K/s**	DHex	23.9	20.0	23.1	20.5	12.5
	ID	2.5	2.1	89.4	5.5	0.5

**Table 4:** EDS data of microstructural features in (CoCrCuTi)_85_Fe_15_ samples.

ɛ (K/s)	Phase	Co	Cr	Cu	Ti	Fe
	Nominal	21.25	21.25	21.25	21.25	15
**~1 K/s**	L1	28.5	23.0	4.6	24.8	19.0
	L2	1.1	0.8	94.9	2.8	0.4
**50–150 K/s**	L1	27.3	24.7	3.5	25.7	18.8
	L2	1.1	1.2	94.8	2.4	0.6
**400–700 K/s**	L1	29.6	23.0	4.4	24.6	18.5
	L2	1.2	0.9	95.5	1.8	0.6
**~1600 K/s**	L1	30.9	20.0	5.6	24.9	18.7
	L2	1.3	1.9	92.7	3.4	0.7

**Table 5:** Vickers hardness and fracture toughness values concerning changes to the cooling rate and composition.

ɛ (K/s)	Composition	Vickers Hardness (HV)	Fracture Toughness (KIC)
	10 at. % Fe	598 ± 129	3.9 ± 0.3
**1 K/s**	12.5 at. % Fe	683 ± 173	4.1 ± 0.2
	15 at. % Fe	623 ± 191	4.1 ± 0.3
	10 at. % Fe	820 ± 35	4.2 ± 0.2
**25** **–** **75 K/s**	12.5 at. % Fe	841 ± 59	3.9 ± 0.3
	15 at. % Fe	891 ± 66	5.5 ± 0.4
	10 at. % Fe	658 ± 119	2.7 ± 0.6
**400–700 K/s**	12.5 at. % Fe	673 ± 92	3.3 ± 0.4
	15 at. % Fe	638 ± 127	2.9 ± 0.5
	10 at. % Fe	790 ± 33	1.7 ± 0.1
**1000 K/s**	12.5 at. % Fe	765 ± 75	1.7 ± 0.4
	15 at. % Fe	760 ± 20	1.9 ± 0.6
	10 at. % Fe	802 ± 22	2.8 ± 0.1
**1600 K/s**	12.5 at. % Fe	877 ± 35	2.6 ± 0.1
	15 at. % Fe	827 ± 53	3.9 ± 0.3

## Data Availability

The original contributions presented in the study are included in the article, further inquiries can be directed to the corresponding author.

## References

[1] Munitz A.B., Kaufman M.J., Nahmany M., Derimow N., Abbaschian R. (2018). Microstructure and mechanical properties of heat treated Al1.25CoCrCuFeNi high entropy alloys. Mater. Sci. Eng. A.

[2] Derimow N., Clark T., Abbaschian R. (2020). Solidification processing and cooling rate effects on hexagonal Co22Cr18Cu20Mn16Ti24 high-entropy alloys. Mater. Chem. Phys..

[3] Derimow N., MacDonald B.E., Lavernia E.J., Abbaschian R. (2019). Duplex phase hexagonal-cubic multi-principal element alloys with high hardness. Mater. Today Commun..

[4] Takeuchi A., Amiya K., Wada T. (2014). High-Entropy Alloys with a Hexagonal Close-Packed Structure Designed by Equi-Atomic Alloy Strategy and Binary Phase Diagrams. JOM.

[5] Derimow N., Jaime R.F., Le B., Abbaschian R. (2021). Hexagonal (CoCrCuTi)100-xFex multi-principal element alloys. Mater. Chem. Phys..

[6] Tsai M.H., Yeh J.W. (2014). High-Entropy Alloys: A Critical Review. Mater. Res. Lett..

[7] Xiong K., Huang L., Wang X., Yu L., Feng W. (2022). Cooling-Rate Effect on Microstructure and Mechanical Properties of Al0.5CoCrFeNi High-Entropy Alloy. Metals.

[8] Miracle D.B., Senkov O.N. (2017). A critical review of high entropy alloys and related concepts. Acta Mater..

[9] Wang F.J., Zhang Y., Chen G.L., Davies H.A. (2009). Cooling Rate and Size Effect on the Microstructure and Mechanical Properties of AlCoCrFeNi High Entropy Alloy. J. Eng. Mater. Technol..

[10] Mishra S.S., Yadav T.P., Srivastava O.N., Mukhopadhyay N.K., Biswas K. (2020). Formation, and stability of C14 type Laves phase in multi-component high-entropy alloys. J. Alloys Compd..

[11] Gorniewicz D., Przygucki H., Kopec M., Karczewski K., Jóźwiak S. (2021). TiCoCrFeMn (BCC + C14) High-Entropy Alloy Multiphase Structure Analysis Based on the Theory of Molecular Orbitals. Materials.

[12] Derimow N.A. (2019). Solidification, Thermodynamics, and Mechanical Properties of Multi-Principal Element Alloys. Ph.D. Thesis.

[13] Li D., Li C., Feng T., Zhang Y., Sha G., Lewandowski J.J., Liaw P.K., Zhang Y. (2017). High-entropy Al0.3CoCrFeNi alloy fibers with high tensile strength and ductility at ambient and cryogenic temperatures. Acta Mater..

[14] Zhang Z., Fu S., Aversano F., Bortolotti M., Zhang H., Hu C., Grasso S. (2019). Arc melting: A novel method to prepare homogeneous solid solutions of transition metal carbides (Zr, Ta, Hf). Ceram. Int..

[15] Chen M.R., Lin S.J., Yeh J.W., Chen S.K., Huang Y.S., Tu C.P. (2006). Microstructure and Properties of Al0.5CoCrCuFeNiTix (x = 0–2.0) High-Entropy Alloys. Mater. Trans..

[16] Singh S., Wanderka N., Murty B.S., Glatzel U., Banhart J. (2011). Decomposition in multicomponent AlCoCrCuFeNi high-entropy alloy. Acta Mater..

[17] Lin C.-M., Tsai H.-L. (2011). Evolution of microstructure, hardness, and corrosion properties of high-entropy Al0.5CoCrFeNi alloy. Intermetallics.

[18] Suárez Ocaño P., Manzoni A., Lopez-Galilea I., Ruttert B., Laplanche G., Agudo Jácome L. (2023). Influence of cooling rate on the microstructure and room temperature mechanical properties in the refractory AlMo0.5NbTa0.5TiZr superalloy. J. Alloys Compd..

[19] Ogura M., Fukushima T., Zeller R., Dederichs P.H. (2017). Structure of the high-entropy alloy AlxCrFeCoNi: FCC versus BCC. J. Alloys Compd..

[20] Zhu C., Xu L., Liu M., Guo M., Wei S. (2023). A review on improving mechanical properties of high entropy alloy: Interstitial atom doping. J. Mater. Res. Technol..

[21] Choi J.W., Kim J.T., Hong S.H., Park H.J., Jumaev E., Kim K.B. (2022). Analysis of phase transformation and deformation behaviors on Laves phase of as-cast (CoCuFeNi)100-xZrx high entropy alloys. J. Alloys Compd..

[22] Ye X., Diao Z., Lei H., Wang L., Li Z., Li B., Feng J., Chen J., Liu X., Fang D. (2023). Multi-phase FCC-based composite eutectic high entropy alloy with multi-scale microstructure. Mater. Sci. Eng. A.

[23] Moradkhani A., Baharvandi H., Tajdari M., Vahdat M., Abedi H. (2013). Determination of fracture toughness using the area of micro-crack tracks left in brittle materials by Vickers indentation test. J. Adv. Ceram..

[24] Kim J., Jung S.-G., Han Y., Kim J., Rhyee J.-S., Yeo S., Park T. (2024). Thermal-driven gigantic enhancement in critical current density of high-entropy alloy superconductors. J. Mater. Sci. Technol..

[25] Zhao Y.J., Qiao J.W., Ma S.G., Gao M.C., Yang H.J., Chen M.W., Zhang Y. (2016). A hexagonal close-packed high-entropy alloy: The effect of entropy. Mater. Des..

[26] Zeng Z., Xiang M., Zhang D., Shi J., Wang W., Tang X., Morita K. (2021). Mechanical properties of Cantor alloys driven by additional elements: A review. J. Mater. Res. Technol..

[27] Chen R., Qin G., Zheng H., Wang L., Su Y., Chiu Y.L., Ding H., Guo J., Fu H. (2018). Composition design of high entropy alloys using the valence electron concentration to balance strength and ductility. Acta Mater..

[28] Farjam R., Akbari A., Nili-Ahmadabadi M., Shirazi H. (2024). Co50Cr20Ni20Fe5Mn5 high entropy alloy: Overcoming the strength-ductility trade-off of Cantor alloy. J. Alloys Compd..

[29] Guo J., Cao C., Gong S., Song R., Bai X., Wang J., Sun Z. (2013). Rapid solidification of Cu60Co30Cr10 alloy under different conditions. Trans. Nonferrous Met. Soc. China.

[30] Sreeramagiri P., Roy A., Balasubramanian G. (2021). Effect of Cooling Rate on the Phase Formation of AlCoCrFeNi High-Entropy Alloy. J. Phase Equilibria Diffus..

[31] George E.P., Curtin W.A., Tasan C.C. (2019). High entropy alloys: A focused review of mechanical properties and deformation mechanisms. Acta Mater..

[32] Nagase T., Mizuuchi K., Nakano T. (2019). Solidification Microstructures of the Ingots Obtained by Arc Melting and Cold Crucible Levitation Melting in TiNbTaZr Medium-Entropy Alloy and TiNbTaZrX (X = V, Mo, W) High-Entropy Alloys. Entropy.

[33] Kozieł T., Pajor K., Gondek Ł. (2020). Cooling rate evaluation during solidification in the suction casting process. J. Mater. Res. Technol..

[34] Wang L., Sun Y., Bo L., Zuo M., Zhao D. (2019). Effects of melt cooling rate on the microstructure and mechanical properties of Al-Cu alloy. Mater. Res. Express.

[35] Luo X., Totten G. (2018). Evolution from Cooling Modeling to Cooling Engineering of the Steel Quenching Process: A Technology Overview. Mater. Perform. Charact..

[36] Pizetta Zordão L.H., Oliveira V.A., Totten G.E., Canale L.C.F. (2019). Quenching power of aqueous salt solution. Int. J. Heat Mass Transf..

[37] Afonso C.R.M., Aleixo G.T., Ramirez A.J., Caram R. (2007). Influence of cooling rate on microstructure of Ti–Nb alloy for orthopedic implants. Mater. Sci. Eng. C.

[38] Elmer J.W. (1988). The Influence of Cooling Rate on the Microstructure of Stainless-Steel Alloys.

[39] Rao K.M.P., Prabhu K.N. (2020). A Comparative Study on Cooling Performance of Hot Oil and Molten Salt Quench Media for Industrial Heat Treatment. J. Materi Eng. Perform..

[40] Žmak I., Ćorić D., Mandić V., Ćurković L. (2019). Hardness and Indentation Fracture Toughness of Slip Cast Alumina and Alumina-Zirconia Ceramics. Materials.

[41] Chicot D., Pertuz A., Roudet F., Staia M.H., Lesage J. (2004). New developments for fracture toughness determination by Vickers indentation. Mater. Sci. Technol..

[42] Samuel A., Prabhu K.N. (2022). Residual Stress and Distortion during Quench Hardening of Steels: A Review. J. Mater. Eng. Perform..

